# Management technology for implementing the Systematization of Nursing Care

**DOI:** 10.1590/1980-220X-REEUSP-2022-0028en

**Published:** 2022-09-02

**Authors:** Anderson Reis de Sousa, George Luiz Alves Santos, Cléton Salbego, Thiago da Silva Santana, Nuno Damácio de Carvalho Félix, Rosimere Ferreira Santana, Rudval Souza da Silva

**Affiliations:** 1 Universidade Federal da Bahia, Escola de Enfermagem, Programa de Pós-Graduação em Enfermagem e Saúde, Salvador, BA, Brazil.; 2 Faculdade Bezerra de Araújo, Rio de Janeiro, RJ, Brazil.; 3 Universidade Federal de Santa Maria, Santa Maria, RS, Brazil.; 4 Universidade Estadual de Feira de Santana, Feira de Santana, BA, Brazil.; 5 Universidade Federal do Recôncavo da Bahia, Santo Antônio de Jesus, BA, Brazil.; 6 Universidade Federal Fluminense, Rio de Janeiro, RJ, Brazil.; 7 Universidade do Estado da Bahia, Senhor do Bonfim, BA, Brazil.

**Keywords:** Biomedical Technology, Nursing Administration Research, Nursing Service, Hospital, Nursing Process, Nurse Practitioners, Tecnología Biomédica, Investigación en Administración de Enfermería, Servicio de Enfermería en Hospital, Proceso de Enfermería, Enfermeras Practicantes, Tecnologia Biomédica, Pesquisa em Administração de Enfermagem, Serviço Hospitalar de Enfermagem, Processo de Enfermagem, Profissionais de Enfermagem

## Abstract

**Objective:**

To describe the construction of a management technology aimed at implementing the Systematization of Nursing Care in nursing services.

**Method:**

This is a methodological, qualitative and explanatory study, based on the normative and legal framework of COFEN Resolution 358/2009. It comprised the theoretical construction of instruments for practice anchored in the literature and expertise of a group of 40 nurses, between April 2020 and June 2021.

**Results:**

The technology is outlined from the dimensions of method, personnel and nursing instruments that support the Systematization of Nursing Care tripod. It consists of an explanatory model of an operational management matrix and a checklist-type instrument for follow-up/monitoring of Systematization of Nursing Care management in services.

**Conclusion:**

Management technology is inserted as a solution to improve organizational performance, health care, clinical decision support, planning, administration, organization of services and professional practice, and create favorable conditions for applying the Process of Nursing at its fullest.

## RESUMO

**Objetivo:**

Descrever a construção de uma tecnologia gerencial voltada à implementação da Sistematização da Assistência de Enfermagem em serviços de enfermagem.

**Método:**

Estudo metodológico, qualitativo e explicativo, sustentado no referencial normativo e legal da Resolução COFEN nº 358/2009. Compreendeu a construção teórica de instrumentos para a prática ancorados na literatura e na *expertise* de um grupo de 40 enfermeiros, entre os meses de abril de 2020 e junho de 2021.

**Resultados:**

A tecnologia está delineada a partir das dimensões método, pessoal e instrumentos de enfermagem que sustentam o tripé da Sistematização da Assistência de Enfermagem. É constituída por um modelo explicativo de uma matriz de gestão operacional e de um instrumento do tipo *checklist* para o acompanhamento/monitoramento da gestão da Sistematização da Assistência de Enfermagem nos serviços.

**Conclusão:**

A tecnologia gerencial se insere como uma solução para melhorar a performance organizacional, a atenção à saúde, o apoio às decisões clínicas, o planejamento, a administração, a organização dos serviços e a prática profissional, e criar condições favoráveis para a aplicação do Processo de Enfermagem em sua plenitude.

## RESUMEN

**Objetivo:**

Describir la construcción de una tecnología de gestión dirigida a implementar la Sistematización de la Atención de Enfermería en los servicios de enfermería.

**Método:**

Estudio metodológico, cualitativo y explicativo, sustentado en el marco normativo y legal de la Resolución COFEN nº 358/2009. Comprendió la construcción teórica de instrumentos para la práctica anclados en la literatura y la experiencia de un grupo de 40 enfermeros, entre abril de 2020 y junio de 2021.

**Resultados:**

La tecnología se perfila a partir de las dimensiones de método, personal e instrumentos de enfermería que sustentan el trípode de la Sistematización de la Atención de Enfermería. Consiste en un modelo explicativo de matriz de gestión operativa y un instrumento tipo checklist para el seguimiento/monitoreo de la gestión de la Sistematización de la Atención de Enfermería en los servicios.

**Conclusión:**

La tecnología gerencial se inserta como una solución para mejorar el desempeño organizacional, el cuidado de la salud, el apoyo a la decisión clínica, la planificación, la administración, la organización de los servicios y la práctica profesional, y crear condiciones favorables para la aplicación del Proceso de Enfermería en su plenitud.

## INTRODUCTION

The problematization around the Systematization of Nursing Care (SNC) is permeated by spaces of conflicts, difficulties of understanding, lack of interest and lack of acceptance by the nursing working class, which is faced with inadequate conditions related to work and professional practice regulation, although the operationalization of SNC in the nursing services is essential for the practice, for the professional identity and for the application of the Nursing Process (NP), in all its stages^(1,2)^.

Based on this scenario, it is necessary to understand the insertion of management technologies (MT) in nurses’ praxis, with a view to systematizing and testing theoretical-practical actions aimed at planning, executing and assessing health processes, aiming at practical interventions to improve quality of care^(3–5)^.

SNC implementation as and through technologies is necessary in Brazileiros health services. Although there are weaknesses, the use of technologies in this process ensures an effective management of customer care in hospital institutions^(6,7)^. Thus, this study is justified to advance the production of scientific knowledge on the problem, describing structuring pillars (method, personnel and instruments) and constituent elements of SNC and proposing a MT for work (re)organization in nursing.

The importance of assuming a current of thought^(7)^ that walks towards the understanding of SNC as a distinct phenomenon from the NP can contribute to the delimitation of the attributes that best characterize it^(8)^, contributing significant implications for professional nursing practice in Brazil^(9,10)^.

There is also that the conceptual confusion between SNC and NP prevents the proper differentiation and application of each of these concepts in practice^(11)^. Therefore, the need for technological innovations with applicability in the daily life of nursing services is urgently needed. Science, through its relations in health, in the involvement with care, education and management actions, needs to keep up to date with regard to promoting discussions and creations about technological development. In this aspect, a trend of advancement can be observed since the mobilization of nurses in a previous period^(3,11)^ until the promulgation of COFEN Resolution (Federal Nursing Council) 272/2002, that provided for SNC, which, at a later time, prompted the movement of its update, resulting in the current COFEN Resolution 358/2009^(12)^. Since then, part of the scientific and technical production on SNC deals with the facilities and difficulties of operationalization in services, but they still need to point out and develop means of its effectiveness in practice^(9,13)^.

In this respect, it is understood that the most appropriate concept to be adopted about what SNC is permeates the organization of professional work in nursing^(14,15)^ in the light of management, based on principles of administration and epistemological elements^(16)^. Thus, this study proposes a technological production that accommodates this epistemological perspective allied to conceptual and operational definitions that provide management opportunities, which are useful to establish the necessary conditions for the organization and/or reorganization of nursing work processes^(4,5)^. Considering the one presented, this study was guided by the research question: what theoretical-conceptual-structural elements should constitute a MT for SNC implementation, in order to support the organization of nursing services? This article aims to describe the construction of a MT focused on SNC implementation in nursing services.

## METHOD

### Study Design and Location

This is a methodological, qualitative, exploratory and descriptive study^(17)^ of development of MT in nursing. The study modeling took place through three stages: (01) literature approach (conceptual and operational structure establishment); (02) prototyping (item construction, selection and organization); (03) final drawing. The proposal construction took place in a remote and participatory way, based on consensus meetings, via Google Meet, among technology developers.

### Selection and Sample Criteria

Specific selection criteria were adopted for the following stages: 01 – literature approach (only studies with convergence to SNC, from the description of method, personnel and instruments published in official sources and in indexed nursing journals). The sample consisted of two stages. The first was composed of documentary data from resolutions and scientific articles. The second stage consisted of qualitative data from self- administered forms, sent to 40 participants. The participants were chosen because they were in practical experience in nursing services at care, training and management levels. Of these, seven were linked to research groups (research nurses) from two public universities in the state of Bahia; 13 were linked to the clinical care area and nursing management; 10 were resident nurses in the emergency area and Intensive Care Unit; and 10 were specialists in the surgical center and robotic surgery area.

### Data Collection

The first stage occurred from a literature review study, conducted based on the Preferred Reporting Items for Systematic Reviews and Meta-Analyses (PRISMAScR), within the reach of national bibliography, available in the MEDLINE/PubMed, CINAHL, Scopus, Web of science core collection, Scielo.org and Latin American and Caribbean Literature on Health Sciences (LILACs) databases, through the Virtual Health Library (VHL). We used the descriptors (DeCS/MeSH) and their respective synonyms, keywords, free terms/additional sources of interest: “Nursing”; “Nursing Care”; “Nurse’s Role”; “Nursing Process”; “Nursing Professional”; “Patient Care Planning”; “Nursing Administration Research”. After the search, all identified citations were pooled and sent to EndNote 20 (Clarivate Analytics, PA, USA). Original articles addressing the concepts of method, personnel and instrument were included. Thus, 18 scientific articles were selected. Duplicate articles were excluded.

A document analysis was also carried out, through a search on COFEN’s website, identifying regulations such as Resolutions (total of 9), which dealt with SNC, subsidized by a specific instrument, previously prepared and validated by the research team. The following were analyzed, both in scientific articles and in documentary sources: title; year of publication; study site; objective; concept (method, personnel and instrument); context (technology development); facilitating and hindering aspects. These stages occurred between April 2020 and August 2021, carried out by a research team (builder committee/inventor group) composed of an undergraduate nursing student, four nurses, one nurse, with a master’s and/or doctoral degree in nursing and health, who worked as university professors and researchers and/or as hospital nurse managers, and professionals in the area of linguistics and diagramming. These are distributed in the five regions of Brazil.

With the elements and respective concepts based on the documentary study, six meetings took place between the authors, for discussions and consensus. The second stage proceeded, with a view to elaborating the theoretical, conceptual and operational structure of MT. This stage was mediated by COFEN Resolution 358/2009. To construct the instruments (prototype), we sought to meet the item behavioral criteria, objectivity, simplicity, clarity, precision, validity, relevance and the interpretability. Thus, by consensus, the inventors developed MT with a structure containing the following aspects: pillar division; conceptual definition; description of items to be surveyed and/or checked in nursing services instituted or in the process of institutionalization. These instruments were organized by consensus among inventors.

### Data Analysis and Treatment

The data obtained from literature approach and nurses’ expertise were treated using the Thematic Content Analysis technique, being operationalized from the following steps: material pre-analysis and exploration; readings and organization of identified findings, as units of meaning, that responded to the object under study, meeting the criteria of exhaustiveness, representativeness, homogeneity, pertinence and exclusivity^(18)^. Finally, there was treatment and interpretation of data based on the normative and legal framework of COFEN Resolution 358/2009, articulated with the prism of nursing management^(12)^. To ensure compliance with the quality of this study, we followed SQUIRE 2.0 recommendations, compatible with the methodological design of the research carried out^(19)^.

### Ethical Aspects

We fulfilled the development stages of this study in relation to ethical aspects. The veracity, reliability, security and quality of the data generated were guaranteed. The study is linked to a matrix research project entitled “*Projeto S@E-Brazil: panorama da sistematização da assistência de enfermagem no território nacional* ”, approved by the Research Ethics Committee, under Opinion 4,746,878/2021. The Resolution 466/12 recommendations were complied with, and the Informed Consent Form was applied for participants’ consent.

## RESULTS

**Figure 1.**
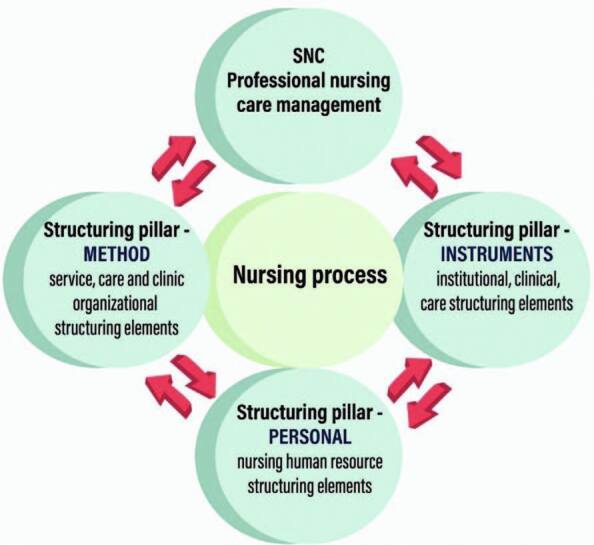
Explanatory/guiding model for implementing the Systematization of Nursing Care in nursing services – Salvador BA Brazil, 2021.

Source: authors.

The instrument structure occurred in three stages, namely: (1) an explanatory model; (2) a management matrix; and (3) a checklist-type instrument for monitoring/monitoring and checking the feasibility and occurrence of the effectiveness of SNC in nursing services, based on administration and management in nursing and health regarding aspects related to implementation, implementation in nursing services. Data from scientific articles and documentary sources were derived from the representative themes for the study (codes) and their respective defining characteristics and constituent elements in terms of properties and dimensions in terms of method, personnel and instruments, which were refined by the inventing team and added and improved, according to participants. Furthermore, the data from participants contributed to the MT design under the following aspects: understanding and intelligibility; usefulness and relevance; facilities and difficulties in use/usability. Furthermore, they referred to the explanatory model structure and form, the constituent elements of the matrix and the instrument-checklist. The explanatory model (1) is represented graphically and imagetically in the SNC structure composition, supported by the legal definition and a theorizing evolutionary movement, which is composed of its structuring pillars (Figure 1).

**Chart 1.**
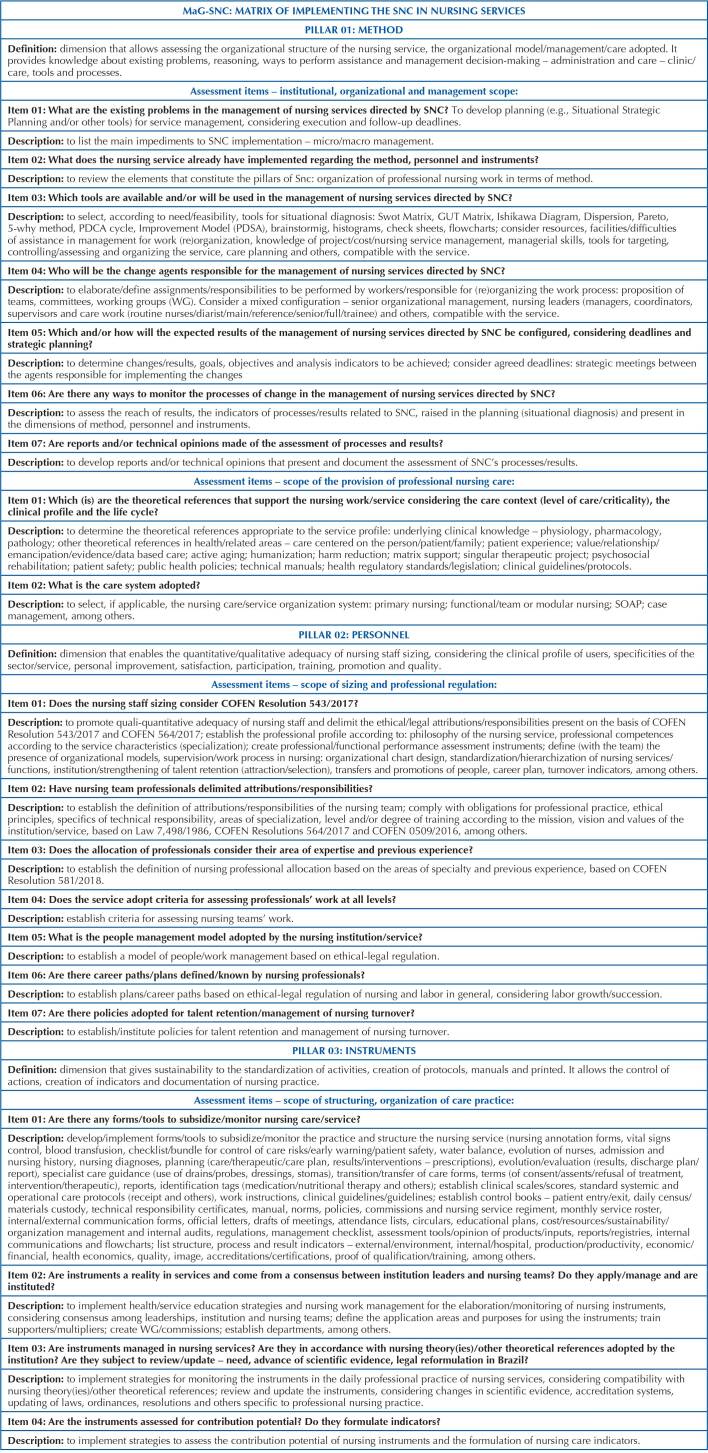
Matrix for implementing the Systematization of Nursing Care in nursing services – Salvador, BA, Brazil, 2021.

Source: authors.

The management matrix (2), presented in Chart 1, for SNC implementation in nursing services, represented by the acronym MaG-SNC, was structured based on the scientific literature on the subject, which made it possible to configure the composition of items and their corresponding descriptions. The parts that compose it are structured by the method, personnel and instruments, according to COFEN Resolution 358/2009, understood as structuring pillars and constituent elements of SNC. Its organization is didactically presented in: definition – conceptualization of each pillar of SNC; assessment items – what needs to be developed/operated/improved by nurse managers; and descriptive – what needs to be organized and/or reorganized for the effectiveness of SNC. A technological innovation in nursing management at MaG-SNC aims to present conceptual and operational approaches to the pillars of SNC as a way of directing nurses and their teams towards the effectiveness of SNC in health services, respecting the reality of the clinical-care profile and the levels of complexity of care in the Brazilian territory.

**Chart 2.**
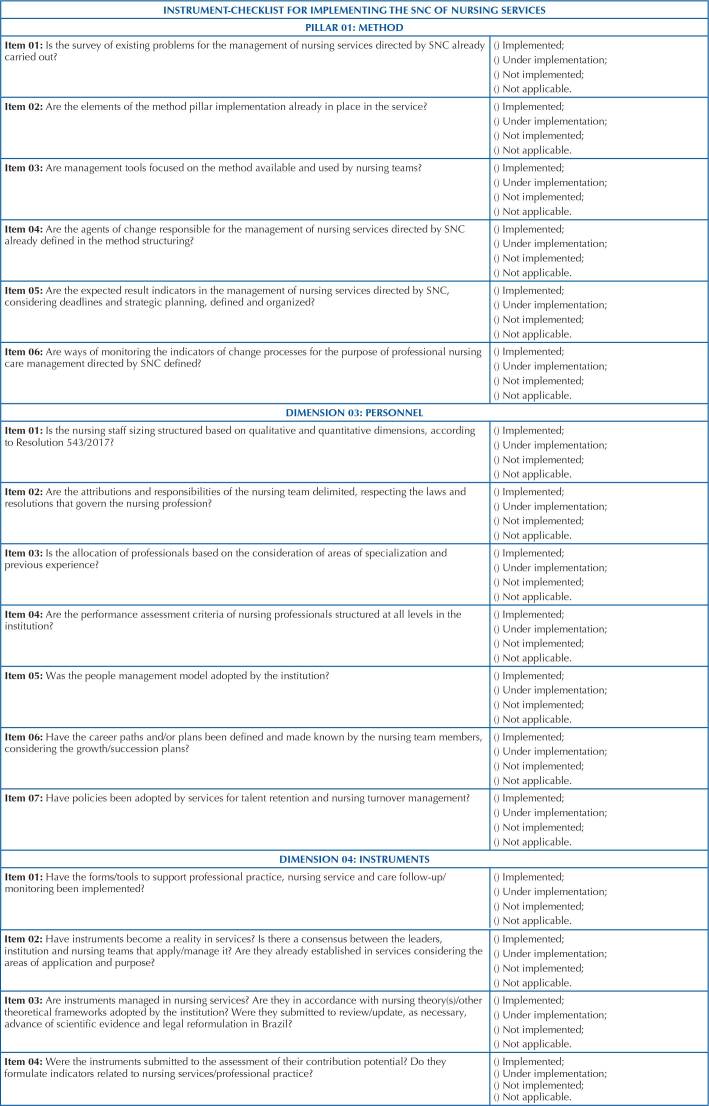
Instrument-checklist for monitoring and assessing SNC implementation in nursing services. Salvador, BA, Brazil, 2021.

Source: authors.

With a matrix for implementing SNC in nursing services – MaG-SNC, which can guide nurses’ action and their teams involved in a project to implement a nursing service and/or in the management of already structured health services, in order to guarantee the institutionalization of SNC, an instrument- checklist was prepared (3) for monitoring and assessment of professional nursing care management directed by SNC. This instrument is organized around the method, personnel and instrument pillars, and is in convergence with the items present in the matrix, and direct nurses and their team to assess the steps of implementing SNC requirements in the nursing services in which it is applied (Chart 2).

## DISCUSSION

This study presents the construction of a MT with guidance in SNC implementation in nursing services. It explained the developmental stages of technology, the technological products produced and their applicability, focus, scope, limits and possibilities, materialized in an operative/directive matrix and a management instrument.

Acting for the management of professional care based on the NP and directed by SNC aims to produce effective and efficient results for the nursing work process, in order to subsidize the audit and assessment of the progress of the operationalization of nursing professional care management directed by SNC in services^(20)^. Thus, the development of technological innovations, as presented in this study, may serve as an elementary subsidy to guide and (re)orient the conduct of nursing service management practices and the strengthening of health service management globally, as reported by the scientific literature, when indicating the expansion of technologies in nursing, especially in terms of health information^(21)^.

Allied to work management, training, education and appreciation of nursing workers, it might favor professional visibility, the expansion of autonomy, governance and governability, scientific, technical and assistance knowledge, as observed in the American scenario. The MT, like the content management for flow optimization, aimed to improve the delivery of quality nursing work nursing care and simplify records^(22)^.

The audit, accreditation and certification processes, especially the improvement of people’s health conditions, are observed as contributions to improving health and nursing indicators, from the perspective of returning to persons/users/demandants/clients or patients and community the existing expressive and symbolic contribution to improve, maintain, rehabilitate, guarantee, repair, recover, reintegrate, restore and restore human integrity and dignity^(15,16)^. In this regard, the Asian context, specifically the Chinese one, has emerged in the advancement of nursing management, from the development of MT, many of them, with the involvement of the Internet of Things (IoT), wireless network applications, barcode technology and infrared sensors, with a focus on improving care work processes^(23)^.

It also provides a significant consultancy device both for nursing professionals, who are positioned at the forefront of decision- making processes for managing services, processes, results, quality, and for nursing workers, who carry out the SNC’s final actions, as well as for the professionals who work in the Federal Council and Regional Councils of Nursing for the education, training, supervision and evaluation of SNC management in services. Such technology palpably materializes the essential elements and requirements for such management to become everyday in services and to be able to resolve doubts between the working category about “what is” and “what is not” SNC^(7,8)^. In this sense, under this situation, the scientific literature has pointed out the lack of MT focused on nursing work, especially in some settings, such as those of acute care production, which involve multiple dimensions and complexities in the daily work of nursing professionals^(24)^. Therefore, it reinforces the usefulness and relevance of our study.

From the management of professional nursing care directed by SNC, through the application of operational technologies, a real contribution is made to work (re)organization, with positive reflexes for nursing workers, when it is sensitive to recognize the specificities, demands, singularities, barriers, vulnerabilities and challenges faced by the category^(16)^. Thus, attention is drawn to the fact that we are not dealing with a romanticized dimension of professional nursing care management directed by SNC, but a political, conscious and rational dimension regarding the daily work process in nursing in hospital care^(7,8)^. Thus, corroborating this perspective, a study carried out in Canada, with nursing managers, pointed out that nursing professionals are the essential agents to facilitate the use of MT, as they are defenders, educators and connectors of the good use of these technologies, which contributes to the linking of the team and resources, professional training and supervision of technological incorporation practices in nursing^(25)^.

That said, we also emphasize that one cannot lose sight of the present need to be guided by the search for improving management of people and human resources in nursing, in the role of macro and/or top management towards valuing the nursing team’s work, and the emergency need to develop and strengthen policies to improve work in health and nursing, which are able to commit to overcoming the (lack of de)value and precariousness of work in this category. Therefore, we emphasize that this technology emerges as a possibility to be analyzed and even used by nursing teams, researchers, formulators of public policies in nursing work in the context of nursing services^(1,2)^. On the other hand, there is an ascending technological growth, in which there is a combination of technological resources: information systems and hospital safety and nursing monitoring; artificial intelligence; nursing platform architectures; database design, for instance, increasingly intuitive and intelligent^(25,26)^.

This context needs to be directed to reflection on the necessary conditions for nursing staff sizing, with a view to transposing the work environment and including ethical and political aspects of the nursing work process, essential for SNC to be implemented^(26)^. Therefore, when the organization of nursing work is compatible and adequate, the benefits to society may be even greater, especially given the fact that nursing professionals may be well positioned to lead critical context management programs, as in Africa, due to HIV control^(27)^, or for improving performance management/performance of Primary Health Care nurses^(28)^. Furthermore, as it constitutes a pillar of SNC, the elements related to human resources in nursing need to be carefully respected so that there is well-being and quality of life at work in nursing, especially in critical contexts, such as a pandemic, which can be better faced through the understanding and practical operation of defining and differentiating contours^(29)^.

Finally, we emphasize that this study emerges as a call for the working category in nursing to also advocate in its favor, with a view to enhancing the political, identity, ethical, salary, scientific, educational and working conditions and career valorization of nursing so that it can “show what it came for”, overcoming and jumping from the place of “automation” and “thingification” of doing, to take the place of the transformation of living instruments and the recognition of the importance in society for “maintenance of life”. As a result, the need to discuss technological development in the field of nursing is emerging and necessary, given the exponential advance of digitalization in health (digital health), as evidenced by an European panoramic study^(30)^.

This study has limitations, which are concentrated in a small group of evaluators, which could possibly bring bias to the technique of measuring information to support the construction of technology in a given historical-temporal frame. However, this study has strengths, as it advances the scientific knowledge available on the subject and provides contributions to practice. This study strengthens the progress regarding the nursing service organization, in order to contribute to NP operationalization and providing professional teams with the necessary tools regarding the work and nursing service organization, in addition to providing added value to the field of nursing management and MT in nursing and health.

## CONCLUSION

The MT is outlined from dimensions method, personnel and instruments that support the pillars of SNC, presenting its epistemological paradigm in the theories of administration and management in health/nursing. It consists of an explanatory model, an operational management matrix and an instrument- checklsit, for the follow-up/monitoring of SNC management in different services.

The findings derived from this study generate contributory implications for nursing science and practice in the care perspective, promoting improvements in organizational performance and reorganizing health/nursing services. Moreover, they provide support to nurses who work in health service management regarding clinical decision-making, implementation of interventions to achieve better results in their practices, increasing quality of care indicators. Thus, in teaching/training and research, given the advance in scientific knowledge on the subject, and from access to a technological product capable of guiding professional practice and enhancing the teaching and service integration based on the popularization of SNC, it improves its processes, bringing visibility and potential to nurses’ work in health/nursing services.
